# Case report: a case of eruptive collagenoma occurring in esophagus and intestine

**DOI:** 10.1186/s13000-017-0617-4

**Published:** 2017-03-20

**Authors:** Ping Gao, Lili Jing, Hai Huang, Cuiping Zhang, Junmei Hao

**Affiliations:** Department of Pathology, Yantai Affiliated Hospital of Binzhou Medical University, No. 717, Jinbu Street, Yantai, Shandong Province 264100 China

**Keywords:** Eruptive collagenoma, Esophagus, Intestine

## Abstract

**Background:**

Eruptive collagenoma is a rare disease. All of the previously reported cases were located on the skin. Here we report such a case occurring in esophagus and intestine.

**Case presentation:**

Our patient is a Chinese woman. Two years ago, hundreds of small nodules were identified in her esophagus and intestine. The lesions were characterized by thickened hyalinized collagen fibers and haphazard neoplastic stellate cells. The tumor cells showed generally positive for vimentin and negative for h-CALD, CD34, desmin, CD163, AE1/AE3, CK7 and CK20. The nodules were blue with Masson Trichrome stain. The clinicopathological, immunohistochemical and histochemical features of the tumor were consistent with eruptive collagenoma. The patient was not given specific treatment after diagnosis, and a routine examination indicated that there was no progress for 2 years.

**Conclusion:**

Hitherto, this is the first case of eruptive collagenoma to have been reported occurring in esophagus and intestine.

## Background

Eruptive collagenoma is a rare acquired connective tissue hamartoma. The first case was described by Cramer in 1966 [1], and a few other cases displaying similar features have been reported since [2–14]. Here, we report such a case occurring in the whole esophagus and intestine. We describe the histopathological features of the lesions as well as the differential diagnoses.

## Case presentation

**Fig. 1:**
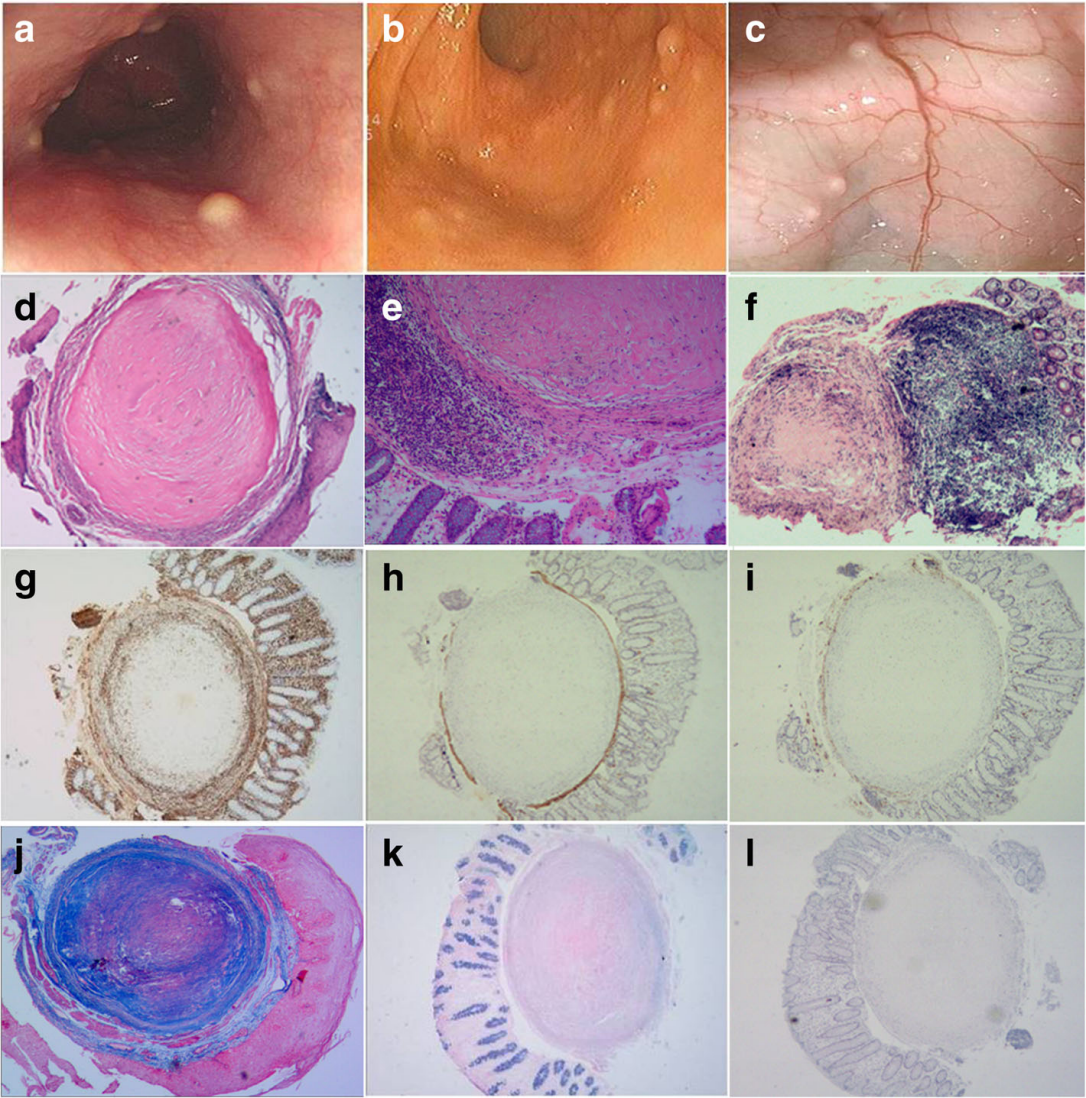
Endoscopy revealed that hundreds of small nodules distributed in esophagus (**a**), terminal ileum (**b**), and colon (**c**). Histologically, several small round nodules located in the submucosa. Most nodules were composed of a large number of collagen and scarce stellate tumor cells (**d**: H&E × 200, **e**:H&E × 400). In some nodules, tumor cells were abundant (**f**: H&E × 200). The tumor cells showed positive for vim (**g**), and negative for h-CALD (**h**, the smooth muscle of muscularis mucosae was positive), CD34 (**i**, the vascular endothelium was positive). The lesions were *blue* with Masson Trichrome stain (**j**, the smooth muscle of muscularis mucosae was *stained red*), *red* with PAS stain (**k**), and *pink* with Congo *red stain* (**l**)

Our patient is a 51-year-old female. Two years ago, she presented with minor abdominal discomfort and dyspepsia for one month. Endoscopy revealed that hundreds of nodules, ranging from 2 mm to 5 mm in diameter, protruded from mucosa in her whole esophagus and intestine (Fig. 1a-c). No ulcer or other abnormal manifestations could be observed. Biopsies were taken from different sites. Laboratory analysis indicated a slightly high IgG level of 25.9 g/L (normal 8.0–17.0 g/L), IgA level of 4.46 g/L (normal 0.72–4.29 g/L) and a slightly low complement C3 level of 0.73 g/L (normal 0.79–1.52 g/L), complement C4 level of 0.14 g/L (normal 0.16–0.38 g/L), and normal ranges for CEA (1.17 ng/ml, normal 0.00–5.00 ng/ml) and AFP (1.40 ng/ml, normal 0.00–8.10 ng/ml). Qualitative check of Bence-Jones protein was negative in urine. The suspected clinical diagnosis was primary systemic amyloidosis, and whether it is a hereditary disease should be differentiated. Her parents and younger sister were all given endoscopy examinations following the patient, but no similar nodules were found in them.

Biopsy tissues from esophagus, terminal ileum, cecum, colon and rectum were submitted for pathological examination. The histopathological characteristics of each specimen were similar. The mucosa was unremarkable, epithelial cells demonstrated bland nuclei, with no atypia or mitotic activity. And no lymphoid inflammatory infiltration in the stroma was observed. In submucosa, there were several small round nodules which were well circumscribed and unencapsulated. The nodules were predominantly composed of collagen bundles, and a variable number of stellate tumor cells located within the collagen (Fig. 1d-f). The collagen were hyalinized and no skeinoid fibers could be observed. The tumor cells were haphazard and not arranged in storiform or palisading pattern. In most nodules, they located peripherally; but in a few nodules, they located diffusely. In all of the lesions, inflammatory cells, calcification, mitoses and necrosis were absent.

Immunohistochemically, the neoplastic cells showed strong cytoplasmic vimentin staining (Fig. 1g), absent h-CALD (Fig. 1h), CD34 (Fig. 1i), desmin, CD163, AE1/AE3, CK7 and CK20 staining. The proliferation rate, measured by detection of ki-67 antigen expression, was very low (approximately positive in 1% tumor cells). Histochemically, the lesions were blue with Masson Trichrome stain (Fig. 1j), red with periodic acid Schiff (PAS) stain (Fig. 1k), and pink with Congo red stain (Fig. 1l). Based on these features, the pathological diagnosis was eruptive collagenoma.

After diagnosis, the patient was given some medicines of proton pump inhibitors and modulating intestinal flora for her symptoms, and no specific treatment was given for collagenoma. One month later, all of the discomfort disappeared. After that, she had a routine endoscopy examination once in 6 months. As of September 2016, no obvious progress was observed.

## Discussion

Collagenomas belong to connective tissue nevi (hamartomas), and are composed predominantly of collagen. They are divided into inherited and acquired. Inherited collagenomas are autosomal dominantly inherited, including familial cutaneous collagenoma and Shagreen patch of tuberous sclerosis. Acquired collagenomas include isolated collagenoma and eruptive collagenoma depending on the number of lesions [15].

Eruptive collagenoma, nevus anelasticus and papular elastorrhexis are closely related entities. Based on the similar clinical and histopathological features, some authors considered they represent a single disease spectrum [3, 16, 17]. They have common features in terms of peak age of onset, distribution of lesions, and a lack of history of trauma. Histologically, the lesion is composed of plump stellate tumor cells, sometimes with interspersed giant multinucleate cells. Romos et al. studied four cutaneous collagenomas using ultrastructural and immunohistochemical analysis. Their findings confirmed that the tumor cells were fibroblastic cells [18]. Collagen deposition was obvious in almost all of the tumors. Occasionally it was the main component of the tumor. In a research published in 2016, Seung et al. evaluated the status of collagen tissue in eruptive collagenoma, nevus anelasticus and papular elastorrhexis. The selected cases were reclassified into three groups: normal collagen group; fine, dense collagen group; and thick, dense collagen group [19]. This study indicated that different kinds of collagen could be observed in collagenomas probably in accordance with different stages of the tumor.

Clinically, all previous cases of eruptive collagenoma were represented with asymptomatic multiple cutaneous nodules. The nodules were skin-colored, dome shaped and usually less than 1 cm in diameter. They occurred mainly on the trunk and upper extremities [2]. The majority age of onset is the first two decades of life. However, there were some cases reported in the later years of life [4, 6, 20]. In our present case, the patient is an old female. Strangely, the lesions were distributed in the submucosa of whole esophagus and intestine. The features of immunohistochemistry were similar to those of eruptive collagenoma reported previously [18]. Masson Trichrome stain confirmed the masses were composed of fibroblastic cells and collagen. And there were no family history and associated disorders. All of these were in favor of the diagnosis of eruptive collagenoma.

The lesions should be differentiated from some other tumors which could be both multinodular and collagenous. Amyloidosis is the first tumor that should be differentiated. It is characterized by abnormal extracellular deposition of specific protein and protein derivatives, which are arranged in a β-pleated sheet structure. There are usually no tumor cells in the lesions. And Congo red stain is red but not pink. These features can distinguish it from eruptive collagenoma. It should also be differentiated from fibromas and plexiform fibromyxoma. Fibromas (fibrous histiocytomas) are ill-defined, characterized by a variable number of spindle and/or rounded cells. A variable admixture of inflammatory cells, coarse collagen bundles in haphazard array are present. Plexiform fibromyxoma (plexiform angiomyxoid myofibroblastic tumor) is a rare benign mesenchymal tumor. The plexiform growth of bland spindle cells in a richly vascularized fibromyxoid stroma is distinctive. GIST is another tumor that should be distinguished. It is the most common primary mesenchymal tumor of the gastrointestinal tract and has a broad morphological spectrum. The tumor cells are usually positive for CD117 and CD34.

So far, the pathogenesis of eruptive collagenoma was not clear. Uitto et al. commented that collagen accumulation is probably associated with a reduced collagenase [21]. Some other investigators considered that hormone may play a role in this disease [5, 22]. Our patient is with a slightly abnormal level of IgG, IgA, complement C3 and complement C4. Whether it is associated with immune system disorder is worthy of further studying.

## Conclusion

We describe a case of eruptive collagenoma occurring in the submucosa of esophagus and intestine, which have never been reported with eruptive collagenoma. We estimate that there are some changes of collagen in the submucosa of gastrointestinal tract that are similar to those on the skin.

## Abbreviation

PAS: Periodic acid Schiff
